# Ocular coherence tomography image data of the retinal laminar structure in a mouse model of oxygen-induced retinopathy

**DOI:** 10.1016/j.dib.2017.09.075

**Published:** 2017-10-06

**Authors:** Wendy A. Dailey, Kimberly A. Drenser, Sui Chien Wong, Mei Cheng, Joseph Vercellone, Kevin K. Roumayah, Erin V. Feeney, Mrinalini Deshpande, Alvaro E. Guzman, Michael Trese, Kenneth P. Mitton

**Affiliations:** a Pediatric Retinal Research Laboratory, Eye Research Institute, Oakland University, Rochester, MI 48309, United States; b Associated Retinal Consultants, Novi, MI, United States; c Control of Gene Expression Laboratory, Eye Research Institute, Oakland University, United States

**Keywords:** Ocular coherence tomography, Retina, Oxygen-induced retinopathy, Mouse retina, Retinal imaging

## Abstract

The data presented in this article are related to the research paper entitled “Norrin treatment improves ganglion cell survival in an oxygen-induced model of retinal ischemia” (Dailey et al., 2017) [1] This article describes treatment with the human Norrin protein, an atypical Wnt-protein, to improve the survival of retinal ganglion cells in a murine model of Oxygen-Induced Retinopathy (OIR). That study utilized Optical coherence tomography (OCT) to visualize retinal layers at high resolution *in vivo*, and to quantify changes to nerve fiber layer thickness. Organization of the laminar structure of other retinal layers in this model *in vivo*, were not known because of uncertainties regarding potential artifacts during the processing of tissue for traditional histology. The OCT image data provided here shows researchers the retinal laminar structural features that exist *in vivo* in this popular mouse OIR model. Traditional H&E stained retinal tissue sections are also provided here for comparison.

**Specifications Table**

**Table:** 

Subject area	*Biology*
More specific subject area	*Oxygen Induced Retinopathy Model of ischemia induced neuron loss.*
Type of data	*Figures of imaging data.*
How data was acquired	*Imaging: Virtual microscopy of retinal histology using fixed tissues with H&E staining. in vivo imaging of retinal layers using Spectral Domain-Optical Coherence Tomography.*
Data format	Images (Tiff format): 1) Bright-field color light microscopy.
	2) Processed SD-OCT images, grey-scale.
Experimental factors	*Young mice were placed into a 70% oxygen environment from age P7 to P12 (five days duration), and then returned to normal oxygen air (21% Oxygen). This created a central retinal zone of vascular ablation during high oxygen exposure, which then experienced aggressive vascular regrowth upon return to normoxia. Eyes received intra-vitreal injections of Norrin or vehicle (PBS).*
Experimental features	*The retinal laminar structure of the same OIR retinas in vivo were captured as OCT data images at two times after neovascular regrowth: post-natal ages P42 and P56.*
Data source location	
Data accessibility	*With this data paper.*

**Value of the data**

• This data shows the laminar structure of the retina in the OIR model *in vivo*.
• This data makes users of the model aware of unique structural changes to the retinal laminar structure *in vivo*.
• The research community can use this data to help determine which changes in traditional histological sections may or may not be artifacts of processing tissue sections.

## 1 Data

**Fig. 1:**
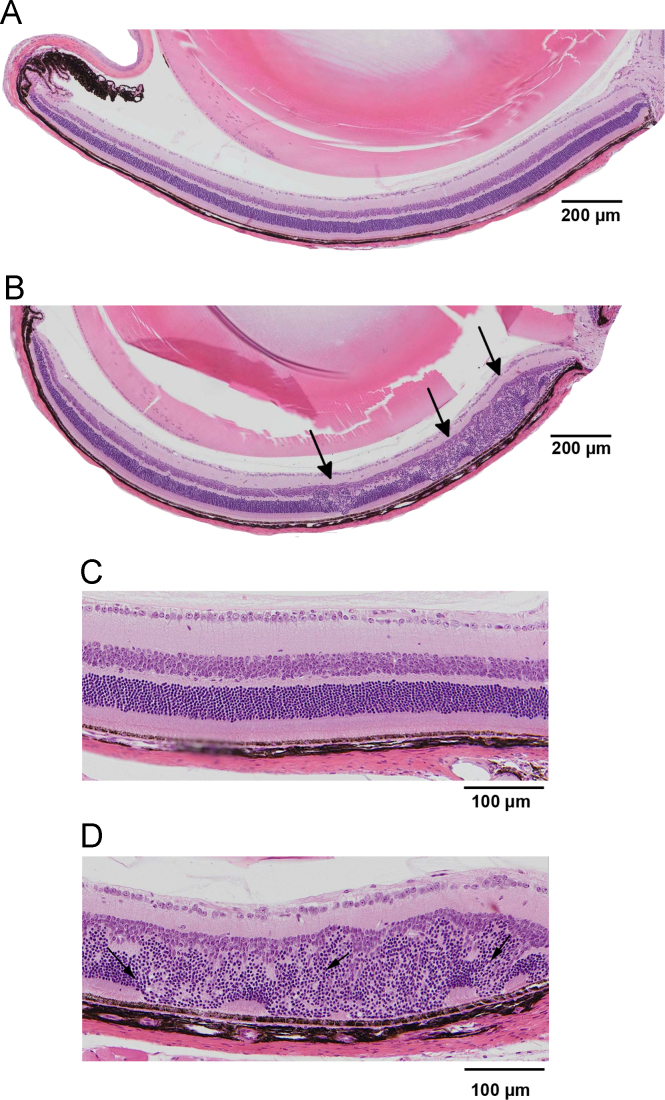
Retinal histology (hematoxylin and eosin staining) of the OIR retina. (**A**) Example of retinal morphology from a room air (normal) mouse, post-natal age 42 days (P42) and (**B**) vehicle-injected OIR mouse (P42) with zones of disruption in the laminar retinal architecture of the central retina (black arrows). (**C**) Zoomed view of the central retina from the same room air (normal) retinal section, showing the characteristically smaller and more densely stained nuclei of the photoreceptor layer. (**D**) Zoomed view of the disrupted central retina from the same vehicle-injected OIR mouse retina shows the location of dense photoreceptor nuclei (black arrows) with nuclei of the inner nuclear layer.

**Fig. 2:**
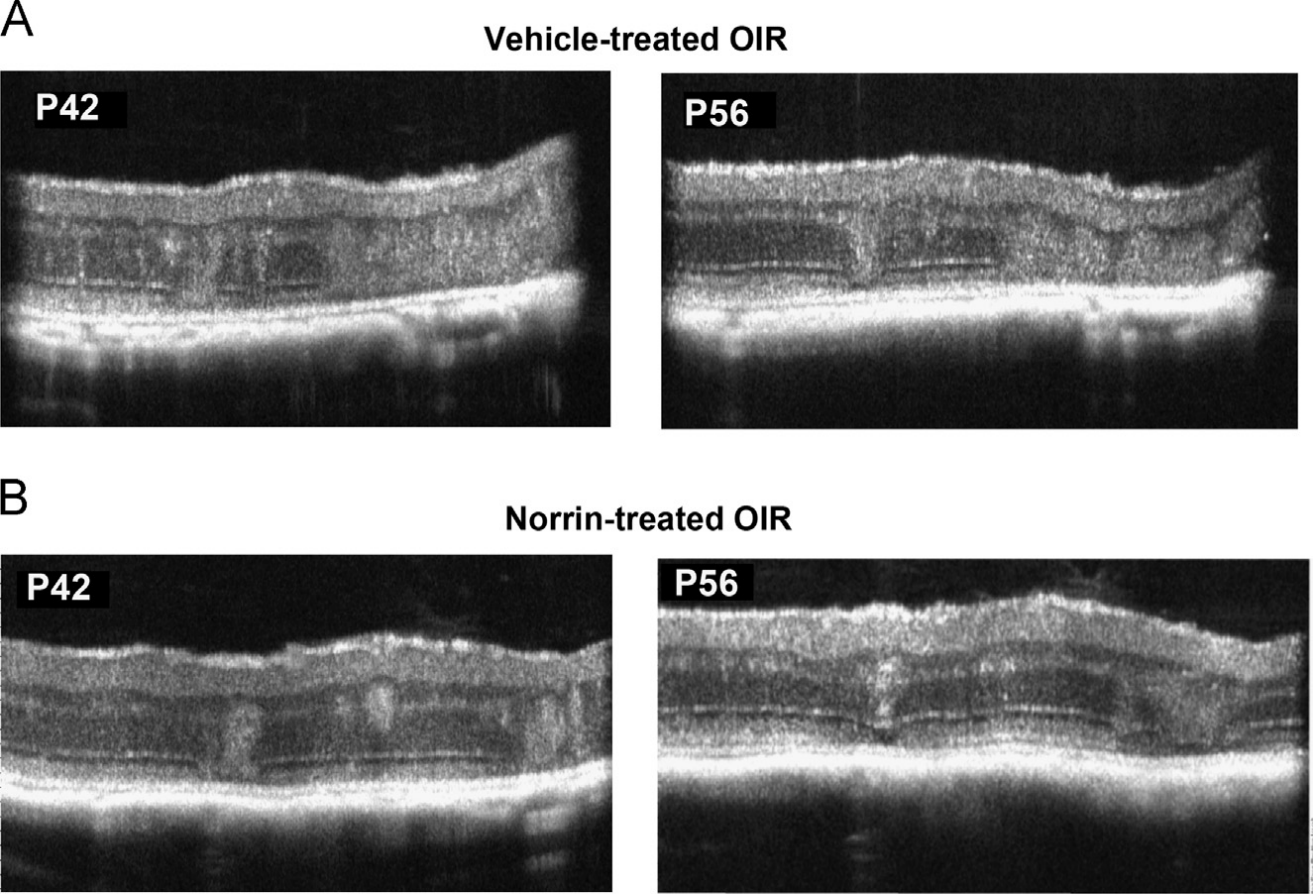
SD-OCT horizontal B-scan data of the retinal laminar structure at ages P42 and P56 in the same OIR retinas. Horizontal B-scans in the superior retina (**A**) Scan of a vehicle-injected OIR eye at ages P42 and P56. (**B**) Scan of a Norrin-injected OIR eye at ages P42 and P56.

This data provides a comparison of traditional H&E stained retinal sections, by bright field microscopy (Fig. 1), with images obtained *in vivo* from living mouse eyes using Spectral Domain Optical Coherence Tomography (SD-OCT) (Fig. 2). Disruptions to retinal layers can be generated during mechanical dissection, fixation, embedding, tissue cutting and the subsequent manipulation of sections during staining. This leaves uncertainty about the cause of any disruptions to the retinal layers that may be seen in traditional histology sections of the retina. The use of SD-OCT provided the ability to examine a popular mouse model of vascular disruption and regrowth, the Oxygen Induced Retinopathy (ORI) model, and this OCT data shows the structural characteristics of the retinal laminar structure at two different time points in the same eyes (42 and 56 days of age).

## 2 Experimental design, materials and methods

### 2.1 Animals

All experiments performed in this study were carried out with the approval of Oakland University's Animal Care and Use Committee and conformed to the ARVO Statement for the Use of Animals in Ophthalmic and Vision Research. Mice were housed at Oakland University in a facility approved by the Association for Assessment and Accreditation of Laboratory Animal Care International. C57BL/6J mice were obtained from Charles River Laboratories (Wilmington, MA).

### 2.2 Oxygen induced retinopathy model

The oxygen-induced retinopathy (OIR) model, using C57BL/6J mice neonates, was used as previously described [1], [2], [3], [4]. This model is well characterized and widely used for experimental studies of retinal ischemia [2]. Mice were exposed to 75% oxygen for five days from ages P7-P12. This high oxygen environment suppresses oxygen-regulated growth factors causing inhibition of vessel growth and vessel loss (vaso-obliteration). At age P12, mice were removed from 75% oxygen chamber and returned to room air (RA).

### 2.3 Intra-ocular norrin injection

Depending on the treatment group, mice received intraocular injections of recombinant human Norrin protein [1]. Norrin (50 ng) or vehicle (1.6 mM HCl in BSS) control was injected into the vitreous cavity of right eyes at age P14.

### 2.4 Virtual microscopy and retinal histology

Using the C57BL/6J strain, room air (RA) and oxygen induced retinopathy (OIR) mice were sacrificed at post natal age of 42 days (P42), their eyes were enucleated and fixed overnight in Davidson's fixative. After paraffin embedding, the eyes were cut into 5 µm sections and stained with Hematoxylin and Eoisin (H&E). The slides were digitized using an Olympus SL120 Virtual Microscopy Slide Scanner (Olympus, Center Valley, PA) with the 20x objective.

### 2.5 SD-OCT analysis of retinal layer thickness

SD-OCT scans were taken using the SD-OCT Envisu R2200 model (Bioptigen, Durham NC), as described in Dailey et al. [1]. A rectangular scan pattern of 1.4 mm × 1.4 mm was used (1000 A-scans by 100 B-scans). In order to capture retinal laminar structural data at more than one time point in the same eyes *in vivo*, SD-OCT was performed at ages P42 and P56 in OIR mice.
